# Cardiorespiratory optimal point as a submaximal evaluation tool in endurance athletes: An exploratory study

**DOI:** 10.3389/fphys.2023.1087829

**Published:** 2023-02-13

**Authors:** Alexis Oyarzo-Aravena, Alexis Arce-Alvarez, Camila Salazar-Ardiles, Rodrigo Ramirez-Campillo, Cristian Alvarez, Camilo Toledo, Mikel Izquierdo, David C. Andrade

**Affiliations:** ^1^ Exercise Applied Physiology Laboratory, Centro de investigación en Fisiología y Medicina de Altura, Departamento Biomédico, Facultad de Ciencias de la Salud, Universidad de Antofagasta, Antofagasta, Chile; ^2^ Magister en Fisiología Clínica de Ejercicio, Facultad de Ciencias, Universidad Mayor, Santiago, Chile; ^3^ Escuela de Kinesiología, Facultad de Odontología y Ciencias de la Rehabilitación, Universidad San Sebastián, Santiago, Chile; ^4^ Navarrabiomed, Hospital Universitario de Navarra (HUN), Universidad Pública de Navarra (UPNA), IdiSNA, Pamplona, Spain; ^5^ Exercise and Rehabilitation Sciences Laboratory, School of Physical Therapy, Faculty of Rehabilitation Sciences, Universidad Andres Bello, Santiago, Chile; ^6^ Department of Physiology, Pontificia Universidad Católica de Chile, Santiago, Chile; ^7^ CIBER of Frailty and Healthy Aging (CIBERFES), Instituto de Salud Carlos III, Madrid, Spain

**Keywords:** musculoskeletal and neural physiological phenomena, exercise test, oxygen consumption, anaerobic threshold, athletic performance, physical endurance

## Abstract

**Introduction:** The cardiorespiratory optimal point (COP) represents the lowest minute ventilation to oxygen consumption ratio (VE/VO2) and can be estimated during a CPET at submaximal intensity when an exercise test until volitional fatigue is not always advisable (i.e., a conflict zone where you cannot be confident of the security because near-competition, off-season, among other). COP’s physiological components have not been wholly described yet. Therefore, this study seeks to identify the determinants of COP in highly trained athletes and its influence on maximum and sub-maximum variables during CPET through principal c omponent analysis (PCA) (explains the dataset’s variance).

**Methods:** Female (*n* = 9; age, 17.4 ± 3.1 y; maximal VO2 [VO2max]), 46.2 ± 5.9 mL/kg/min) and male (*n* = 24; age, 19.7 ± 4.0 y; VO2max, 56.1 ± 7.6 mL/kg/min) athletes performed a CPET to determine the COP, ventilatory threshold 1 (VT1) and 2 (VT2), and VO2max. The PCA was used to determine the relationship between variables and COP, explaining their variance.

**Results:** Our data revealed that females and males displayed different COP values. Indeed, males showed a significant diminished COP compared to the female group (22.6 ± 2.9 vs. 27.2 ±3.4 VE/VO2, respectively); nevertheless, COP was allocated before VT1 in both groups.

**Discussion:** PC analysis revealed that the COP variance was mainly explained (75.6%) by PC1 (expired CO_2_ at VO2max) and PC2 (VE at VT2), possibly influencing cardiorespiratory efficiency at VO2max and VT2. Our data suggest that COP could be used as a submaximal index to monitor and assess cardiorespiratory system efficiency in endurance athletes. The COP could be particularly useful during the offseason and competitive periods and the return to the sports continuum.

## Introduction

It has been proposed that the more important physiological determinants of exercise performance in all-out performances lasting 21–60 min are maximal oxygen consumption (VO2max), lactate threshold, and movement economy (Midgley et al., 2007). Physiological determinants are routinely assessed through a maximal cardiopulmonary exercise test (CPET); however, a CPET may be problematic under specific athletic scenarios, such as after a period of training cessation or injury. Of note, 66%–91% of athletes return to sports activities after a lower extremity surgery, while a return to pre-injury level performance is possible in 31%–68% of all cases (Vereijken et al., 2020). The strategic assessment of risk and risk tolerance proposes a three-step model to help return to sports decision-making (Shrier, 2015). Particularly, step 2 of the functional test estimates the stress and how much stress can tolerate the injured tissue (Shrier, 2015). However, this strategy was designed to evaluate the functional capacity of the joints and bones. In contrast, cardiorespiratory capacity evaluation throughout a maximal effort test could be problematic in injured athletes during the early stages through the return to sport continuum (Shrier, 2015; Ardern et al., 2016).

Besides, considering injuries (Shrier, 2015) and detraining periods (Mujika and Padilla, 2000a; Mujika and Padilla, 2000b), it could be helpful to evaluate exercise performance during these training cessations. Classically, endurance performance is evaluated through a CPET, and ventilatory thresholds (VTs) have been used to delineate moderate to heavy exercise intensity domains; non-etheless, their reliability is questionable due to the multiple methods used to obtain those calculations (Jamnick et al., 2020). Exercise intensity domains have been useful in manipulating training intensity in athletes who perform close to 80% of their training at low-intensity (BILLAT et al., 2003). Sandbakk et al. (2011) reported that world-class skiers performed 30% more low and moderate-intensity training than national-class skiers. Therefore, reliable submaximal measurements or tests could benefit athletes, providing helpful information to normalize exercise intensity (Sandbakk et al., 2011), which is needed for physiological adaptations to achieve improved performance (Midgley et al., 2007). Hence, it is necessary to track performance-related variables or even to estimate the cardiorespiratory maximum values through submaximal tests, which could help to know how the cardiorespiratory system of endurance athletes could be affected during the off-season and competitive periods, but without the need to perform the maximal effort.

The cardiorespiratory optimal point (COP) is an index obtained/calculated from a submaximal CPET, capable of evaluating the interaction between cardiovascular and respiratory systems through VO2 efficiency (Ramos et al., 2012). COP has been defined as the lowest minute ventilation VE/VO2 ratio in a given minute during a CPET (Ramos et al., 2012). Commonly, the COP is located before the first ventilatory threshold (VT1) (Ramos et al., 2012; Ramos and Araújo, 2017; Silva et al., 2018) and has been interpreted as the lowest VE required to extract 1 L of oxygen, which showed the efficiency of the breathing response to consume oxygen during exercise (Ramos et al., 2012; Ramos and Araújo, 2017; Silva et al., 2018). Importantly, it has been shown that COP could predict the mortality risk independent of sex and comorbidities (Ramos and Araújo, 2017), and to assess the exercise performance of professional soccer players (Silva et al., 2018). Of note, preliminary evidence suggests no correlation of COP with ventilatory thresholds (VTs) or VO2max (Silva et al., 2018; Charitonidis et al., 2020). Nevertheless, the reported absence of correlation might not be able to explain a possible influence and COP variance on multiple combinations of cardiovascular, respiratory, and metabolic variables. In addition, the COP, due to its location at low-intensity (Ramos et al., 2012; Ramos and Araújo, 2017; Silva et al., 2018), could be a helpful tool for estimating and monitoring changes in metabolic and cardiorespiratory variables associated with high-performance variables at a low time-effort ratio in athletes who perform >80% of their training at low-intensity (Seiler, 2010; Stöggl and Sperlich, 2015). Principal Component Analysis (PCA), a statistical method applied to data to reduce the number of variables in principal components, could explain the most variance of specific data helping to determine critical variables (Ringnér, 2008). Thus, considering that there is no compelling evidence on the determinants of COP and how it affects cardiorespiratory performance variables; the present study aimed to seek the determinants of COP in highly trained athletes and its influence on maximum and sub-maximum variables during CPET through PCA.

## Methods

### Experimental approach to the problem

We performed a study to assess the COP in highly trained athletes (McKay et al., 2022) during an incremental CPET trial and its influence on maximum and sub-maximum variables through PCA. The CPET was performed on a cycle ergometer. In addition, resting metabolic rate and pulmonary function were determined to gather control variables required for enrollment. The PCA was performed with 72 continuous numerical variables obtained during CPET.

### Participants

Thirty-three highly trained endurance athletes (female, *n* = 9; male, *n* = 24) voluntarily participated in this study. All-female participants were measured in the same stage of the menstrual cycle in different weeks. Exercise testing sessions were conducted between 08:00 and 17:00 h. Previously, all participants and parents of underage athletes were carefully informed about the experiment procedures and the possible risks associated with their participation in the study. They were instructed to refrain from consuming drugs, ergogenic aids, foods, or substances that alter autonomic control or sports performance 48 h before the maximum exercise test. A signed informed consent or assent document was obtained from parents and/or legal guardians, a document that attests to informed consent from a parent and/or legal guardian for study participation that is in accordance with the latest version of the Declaration of Helsinki. All protocols were evaluated by the ethical committee of Universidad Mayor (#169_2019).

### Experimental procedure

All participants had a background in endurance activities (medium and long-distance swimmers, road bikers, and long-distance runners) and were part of the regional team (train >3 h per day, 6 days per week, and minimum 5 years of training with a background in national competitions, being classified in Tier three or “Highly Trained/National Level” according to the Participant Classification Framework (McKay et al., 2022). Exclusion criteria considered for enrollment were: (i) potential medical problems or a history of ankle, knee, or back injury; (ii) any lower extremity reconstructive surgery in the past 2 years or unresolved musculoskeletal disorders; (iii) history of chronic obstructive or restrictive pulmonary diseases and/or altered spirometry on the day of the pre-exercise session (forced expiratory volume at first second (FEV1)/vital capacity (VC) <70, FEV1<80% of predicted value or VC < 80% of predicted value). Inclusion criteria were: i) Being part of the regional team with a background in endurance performance, ii) absence of cardiopulmonary or electrocardiogram (ECG) alterations related to the disease or autonomic dysfunction (as an indicator of overtraining [data not showed]). Participants were familiarized with the test procedures before the measurements were taken. All participants were subject to the same warm-up muscle actions before the exercises (Andrade et al., 2015). The coaches were asked to give the athletes 24 h of rest, and the day before each experimental condition, participants were instructed to (i) have a good night’s sleep (∼8 h) and (ii) use the same athletic shoes and clothing during the protocols.

Prior to CPET, height, body mass, ECG, and clinical spirometry (VC; peak expiratory flow [PEF]; peak inspiratory flow [PIF]; FEV1; FEV1/VC; forced expiratory flow at 25% [FEF 25], 50% [FEF 50] and 75% [FEF 75] of VC) were taken. Height was measured using a wall-mounted stadiometer (HR-200, Tanita, Japan) to the nearest 0.1 cm. Body mass was measured to the nearest 0.1 kg using a digital scale (BF-350, Tanita, IL, United States). BMI was calculated as body mass/height2.

### Cardiopulmonary exercise test (CPET) and cardiorespiratory optimal point

Exercise testing was supervised by an experimented technician according to the American Thoracic Society Guidelines (Weisman et al., 2003). All participants performed the CPET according to the modified Astrand ramp protocol using Convival CPET cycle ergometer (Lode, Netherlands). The cycle ergometer seat was set for each participant (e.g., seat and bar height), prior to each testing session. CPET was performed to determine COP, VO2max, and ventilatory threshold 1 (VT1) and 2 (VT2) and was similar to what has been previously described (Beaver et al., 1986). Briefly, before the maximal test, the participants had a rest time of 5 min on the cycle ergometer, then performed 5 min of warm-up and at an intensity of 25 W. The test started at 50 W, and the workload was increased by 25 W/min until they could not maintain the prescribed cycling frequency of 70 rpm for more than five consecutive seconds (Fletcher et al., 2013). During the test, participants breathed through a valve (Hans Rudolph, United States), and for expired and inspired gas collection and analysis, the Quark CPET metabolic cart (COSMED, Italy) was used. The COP was defined as the lowest oxygen ventilatory equivalent value (VE/VO2 ratio), obtained from an average of six 10-s windowing samples in a given minute, similar to what has been previously described (Ramos et al., 2012). Before each trial, the system was calibrated with a mixture of O2 and CO_2_ known (O2 15%, CO_2_ 5%, N2 balanced; Carburos Metálicos, Barcelona, Spain). Flowmeter calibration was performed using a certified 3 L calibration syringe. VT1 was in the VCO_2_ vs. VO2 panel when the intersection between the two linear segments occurs (Beaver et al., 1986) when VE/VO2 begins to increase after being constant or slightly decreasing while VE/VCO_2_ has been flat or slightly decreasing (Wasserman, 1984). VT2 was in VE vs. VCO_2_ panel when the intersection between the two linear segments occurred (Beaver et al., 1986) with an increase in both VE/VO2 and VE/VCO_2_ (Wasserman, 1984).

### Pulmonary function

Pulmonary functions were assessed according to both the American Thoracic Society and the European Respiratory Society consensus and similar to what has been previously described. Briefly, all participants were asked to exert maximum effort during forced breathing. Results were derived from three repeated measurements, with between-maneuver variation <5% or 200 mL in forced vital capacity (FVC) and forced expiratory volume at first second (FEV1). The maximal mid-expiratory flow was selected from the best maneuver, that is, the maneuver with the largest sum of FVC and FEV1. We used the maximal expiratory curve to calculate FVC, PEF, PIF, FEV1, FEV1/VC, FEF 25, FEF 50, and FEF 75 of VC. All recordings were performed using Quark PFP spirometer (COSMED, Italy).

### Resting metabolic rate

The resting metabolic rate (RMR) was performed by indirect calorimetry using Quark CPET metabolic cart (COSMED, Italy); accordingly, to has been previously described. Briefly, the participants were instrumentalized with an oronasal mask (7450 Series Silicone V2, Hans Rudolph, Kansas City, United States) for expired and inspired gas collection and analysis (Quark CPET metabolic cart; COSMED, Roma, Italy). Nevertheless, before the measurement, the participant took a rest of 30 min in supine positions. After, the recording started, the total time was 40 min, where the first 5-min were discarded as part of the acclimatization period, and the calculation of respiratory quotient (RQ), protein oxidation, carbohydrates, and lipids were calculated from the remaining 35 min. Protein oxidation, carbohydrates, and lipids were expressed as kcal/day and as % of the total resting metabolic rate. The RMR measurement was performed in a specially conditioned room isolated from noise at a temperature of 23°C and 50% of humidity. The RMR was evaluated between 8:00 to 10:00 a.m. For every three measurements, the metabolic cart was re-calibrated with a known calibration gas (O2 15%, CO_2_ 5%, N2 balanced). The recording and analysis were performed with OMNIA, Cardiopulmonary Diagnostic Suite v 1.4 (Quark CPET metabolic cart; COSMED, Roma, Italy).

### Statistical analysis

Data are presented as mean ± standard deviation. Normality (Shapiro-Wilk test) and homoscedasticity (Levene test) tests were performed. To compare both groups, unpaired t-test or Mann-Whitney tests were performed accordingly to data distribution. The α level for all statistics was set as *p* < 0.05. All statistical calculations were performed by GraphPad Prism 9.0 (GraphPad software Inc, CA, United States).

### Multivariable correlations and principal component analysis (PCA)

To determine the contribution of different maximal and submaximal variables to explain the COP, we used a PCA to define groups of variables, which could explain the COP variance (Di Carlo et al., 2015). Data from all subjects were organized into an “n” x “m” matrix (with no missing entries), with “n” rows indicating observations (subjects) and “m” columns representing cardiorespiratory, metabolic, and morphological variables (dimensions), generating a 72 × 72 matrix. A Heatmap of the correlation matrix was generated from computed Pearson-r values for every pair of datasets using GraphPad Prism software v9 (GraphPad software Inc, CA, United States). Only numerical continuous variables are considered for multivariable analysis and dimensional reduction, and detailed information of Pearson-r values and the variables used for analysis are depicted in Supplementary Table S1.

PCA was performed after data standardization of the 72 × 72 matrix and eigenvalue decomposition. Data standardization was performed by computing z-scores according to the formula:

$$zi=\frac{\left(xi-\mu \right)}{\sigma }$$

where zi corresponds to the z-score of every individual value, xi to raw individual values, and µ and σ to the mean and standard deviation of datasets, respectively (Jolliffe et al., 2016). Data standardization, eigenvalues, component loadings, and PC scores were calculated using GraphPad Prism software v9 (GraphPad software Inc, CA, United States). For PCA, only the eigenvalues higher than one were considered as significant, according to the Kaiser’s rule (Kaufman and Dunlap, 2000). The first component (PC1) accounts for most of the total variance of data, and it is associated with the largest eigenvalue; PC2 accounts for as much as possible of the remaining variance, and so on (Di Carlo et al., 2015). Component loadings are equal to a Pearson correlation between the principal component, and a quantified variable is a set of optimal weights (Supplementary Table S2). PC scores were plotted in a biplot to display the information of all individuals as points in the same space as the variables (Di Carlo et al., 2015). After PCA, biplots were created to illustrate the relationship between the variables in the space of selected components.

## Results

### Baseline physiological variables

**FIGURE 1:**
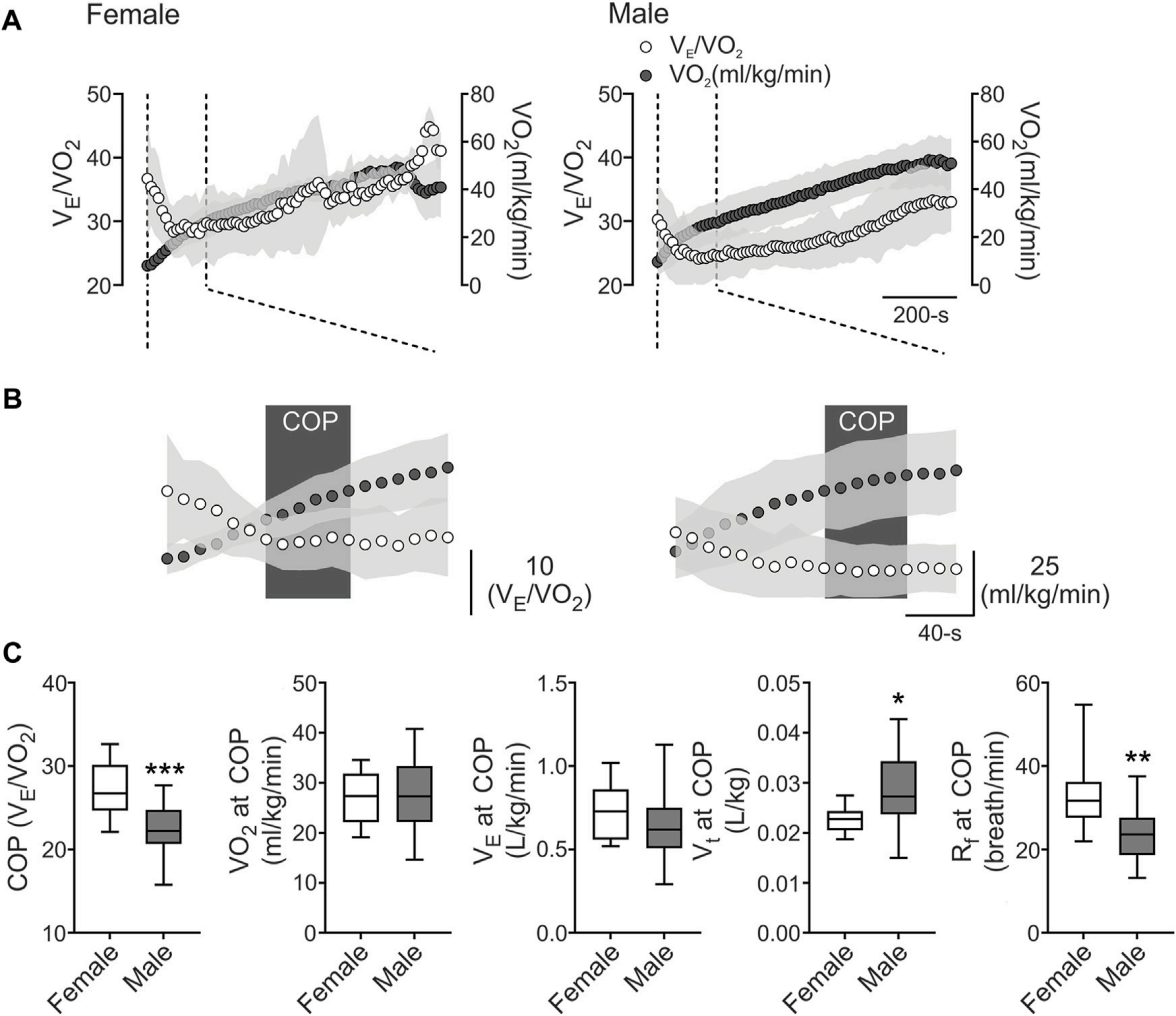
The cardiorespiratory optimal point between females and males. **(A)**, Representative VE/VO2 and VO2 recording from one female and one male well-trained athlete. **(B)**, Representation of cardiorespiratory optimal point (COP) determined by minute ventilation/oxygen consumption (VE/VO2). Note that males displayed a lower COP value compared to female participants. **(C)**, Summary data of COP and VO2, VE, tidal volume (VT), respiratory frequency (Rf) at COP (from left to right, respectively). Male participants displayed a higher COP and VO2 at COP and VT compared to female athletes. Also, males displayed a decrease in Rf compared to females. Unpaired T-test, *, *p* < 0.05; **, *p* < 0.01; and ***, *p* < 0.001. Female, *n* = 9; and Male, *n* = 24.

Baseline physiological data of demographic, cardiopulmonary, metabolic, and pulmonary function are shown in Table 1. All athletes reached VO2max over their predicted values, and pulmonary function showed normal volumes and capacities of the lungs (Table 1). Our data revealed that COP was located before VT1 (Figures 1A, B), and the COP value in male and female athletes was between 22 and 30 VE/VO2 ratio (22.64 ± 2.95 vs. 27.24 ± 3.36 VE/VO2, respectively) (Figures 1B, C). The cardiorespiratory and metabolic data at COP are shown in Table 2. After VTs and VO2max identification, we assessed cardiorespiratory and metabolic variables during these stages, which are depicted in Table 3.

**TABLE 1:** Demographics, cardiopulmonary, resting metabolic rate, and pulmonary function.

		Female (*n* = 9)	Male (*n* = 24)
Characteristics			
	Age (years)	17.44 ± 3.13	19.71 ± 4.01
	Height (cm)	160 ± 5.59****	175.4 ± 9.04
	Weight (Kg)	56.62 ± 7.14**	69.92 ± 12.88
	BMI	22.12 ± 2.69	22.56 ± 2.67
Cardiopulmonary			
	VO2max (ml·kg-1·min-1)	46.17 ± 5.87**	56.05 ± 7.57
	VO2pred (%)	125.9 ± 14.53	133.2 ± 20.03
	RER	1.161 ± 0.12	1.185 ± 0.08
	VT1 (ml·kg-1·min-1)	30.03 ± 4.85**	38.17 ± 7.33
	VT1%VO2max	65.33 ± 8.99	68 ± 9.18
	VT2 (ml·kg-1·min-1)	37.43 ± 5.81**	47.2 ± 8.08
	VT2%VO2max	81.44 ± 8.97	84.25 ± 9.84
Resting metabolic rate			
	RMR (Kcal/day)	1834 ± 390**	2348 ± 362.4
	FAT%	58.94 ± 20.49	55.35 ± 25.06
	CHO%	41.06 ± 20.49	44.65 ± 25.06
Spirometry			
	FVC (L)	3.803 ± 0.55****	5.444 ± 1.04
	FVCpred (%)	108.6 ± 10.08	105.9 ± 9.73
	FEV1 (L)	3.299 ± 0.5****	4.515 ± 0.72
	FEV1pred (%)	105.8 ± 10.92	102.4 ± 10.06
	PEF (L/s)	6.517 ± 1.02***	8.684 ± 1.4

Values are shown as mean ± standard deviation. BMI: body mass index; VO2max: maximal oxygen consumption; VO2pred: Predicted maximal oxygen consumption; RER: respiratory exchange ratio; VT1: ventilatory threshold one; VT1%VO2max: distance from VT1 to VO2max in percentage; VT2: ventilatory threshold 2; VT2%VO2max: distance from VT2 to VO2max in percentage; RMR: resting metabolic rate; FAT%: percentage of energy from fat; CHO%: percentage of energy from carbohydrate; FVC: forced vital capacity; FVCpred: predicted forced vital capacity; FEV1: forced expiratory volume; FEV1pred: predicted forced expiratory volume in 1 s; PEF: peak expiratory flow. *p < 0.05; **p < 0.01; ***p < 0.001 and ****p < 0.0001.

**TABLE 2:** Cardiopulmonary exercise test variables at COP.

	Female (*n* = 9)	Male (*n* = 24)
RfCOP	23.36 ± 5.90	33.27 ± 9.16***
VTCOP	1.914 ± 0.49	1.300 ± 0.28**
IVCOP (L)	1237 ± 257.7	1836 ± 494.2 **
VO2COP	1,911 ± 451.7	1,534 ± 326.7*
RERCOP	0.8386 ± 0.05	0.8148 ± 0.09
VE/CO2COP	32.61 ± 4.6	27.86 ± 2.92 **
HRCOP (beats·min-1)	135.5 ± 9.47	120.8 ± 15.88 **
VO2/HRCOP (ml·beats-1)	11.27 ± 2.1	15.82 ± 3.22 ***
PetO2 (mmHg)	96.92 ± 4.66	89.93 ± 5.76 **
PetCO2 (mmHg)	37.47 ± 4.98	42.01 ± 4.09 **
FAT%COP	54.96 ± 15.76	62.78 ± 28.27
CHO%COP	45.04 ± 15.76	37.22 ± 28.27
TiCOP (s)	0.8541 ± 0.158	1.284 ± 0.37 ****
TeCOP (s)	1.086 ± 0.33	1.498 ± 0.45 **
TtotCOP (s)	1.939 ± 0.47	2.782 ± 0.81 **
Ti/TtotCOP	0.4466 ± 0.03	0.4621 ± 0.02
VD/VTCOP	0.1953 ± 0.02	0.184 ± 0.02
VT/TiCOP	1.536 ± 0.22	1.566 ± 0.43

Values are shown as mean ± standard deviation. RfCOP: respiratory frequency at COP; VTCOP: tidal volume at COP; VECOP: ventilation at COP; IVCOP: inspiratory volume at COP; VO2COP: absolute oxygen consumption at COP; RERCOP: respiratory exchange ratio at COP; VE/CO2COP: ventilatory equivalent for carbon dioxide at COP; HRCOP: heart rate at COP; VO2/HRCOP: oxygen pulse at COP; PetO2: end tidal partial pressure of oxygen at COP; PetCO2: end tidal partial pressure of carbon dioxide at COP; FAT%COP: percentage of energy from fat at COP; CHO%COP: percentage of energy from carbohydrate at COP; TiCOP: inspiratory time at COP; TeCOP: expiratory time at COP; TtotCOP: respiratory cycle duration at COP; Ti/TtotCOP: inspiratory time divided by respiratory cycle duration at COP; VD/VTCOP: dead space at COP; VT/TiCOP: ventilatory drive at COP. *p < 0.05; **p < 0.01; ***p < 0.001 and ****p < 0.0001.

**TABLE 3:** Sex-related cardiopulmonary differences.

	VT1						VT2							VO2max						
Variables	Female			Male			Female			Male				Female				Male		
t%	44.67	±	9.1	50.25	±	16.08	68.22	±	12.23	74.92	±	13.42	---			---	----			---
Rf	36.09	±	9.57	29.69	±	7.09*	41.89	±	8.72	36.33	±	7.86	52.46		±	9.86	51.18		±	7.63
VT	1.367	±	0.25	2.266	±	0.5****	1.572	±	0.17	2.56	±	0.52****	1.86		±	0.23	2.8		±	0.52****
VE	47.07	±	7.63	64.37	±	10.57****	64.76	±	10.52	90	±	16.08***	96.47		±	19.58	140.6		±	23****
IV	1318	±	226.6	2256	±	454.2****	1538	±	164.7	2575	±	486.2****	1863		±	249.2	2860		±	517.6****
VO2	1687	±	269	2624	±	477****	2106	±	278.3	3251	±	539.7****	2599		±	373.6	3863		±	520.4****
VCO_2_	1468	±	212.6	2421	±	487.2****	2052	±	308.4	3322	±	692.9****	2885		±	369.7	4406		±	660.9****
RER	0.8721	±	0.05	0.92	±	0.056*	0.9752	±	0.08	1.017	±	0.07	1.12		±	0.08	1.14		±	0.07
VE/VO2	28.1	±	3.58	24.74	±	2.4**	31.06	±	5.3	27.79	±	2.89*	37.66		±	9.14	36.41		±	3.54
VE/VCO_2_	32.33	±	4.66	27.02	±	3.31***	31.9	±	5.03	27.42	±	3.15**	33.65		±	6.63	32.02		±	3.46
METs	8.58	±	1.39	10.91	±	2.09**	10.72	±	1.62	13.49	±	2.31**	13.19		±	1.68	16.01		±	2.17**
HR	145.9	±	13.1	146.4	±	13.93	170	±	9.46	169.7	±	15.33	186.9		±	5.47	189.5		±	9.62
VO2/HR	11.58	±	1.74	17.99	±	3.17****	12.44	±	1.9	19.24	±	3.07****	13.94		±	2.15	20.45		±	2.92****
PetO2	97.73	±	4.8	93.34	±	4.2*	100.6	±	4.94	96.95	±	4.13*	105.2		±	4.92	104.4		±	3.17
PetCO2	37.86	±	4.9	43.56	±	4.61**	38.39	±	5.21	43.39	±	4.27**	37.21		±	5.48	38.83		±	3.65
FAT	5202	±	2387	4953	±	3235	2468	±	2647	1576	±	2456	114		±	245.3	135.9		±	630.3
CHO	6598	±	1856	13617	±	4628****	12613	±	3000	21944	±	5149****	19054		±	2433	28536		±	3759****
FAT%	43.63	±	16.21	27.84	±	18.28*	16.15	±	16.94	7.265	±	11.12	0.5		±	1.03	0.3512		±	1.6
CHO%	56.37	±	16.21	72.16	±	18.28*	83.85	±	16.94	92.74	±	11.12	99.5		±	1.03	99.65		±	1.6
Ti	0.8054	±	0.17	1.015	±	0.23*	0.6928	±	0.11	0.85	±	0.2*	0.57		±	0.09	0.59		±	0.1
Te	0.9854	±	0.33	1.124	±	0.26	0.8035	±	0.2	0.9	±	0.22	0.62		±	0.14	0.63		±	0.1
Ttot	1.791	±	0.48	2.139	±	0.47	1.496	±	0.3	1.74	±	0.41	1.19		±	0.22	1.21		±	0.19
Ti/Ttot	0.4565	±	0.03	0.4747	±	0.02	0.4675	±	0.03	0.49	±	0.03	0.48		±	0.03	0.49		±	0.02
VD/VT	0.1981	±	0.02	0.1906	±	0.02	0.2037	±	0.03	0.2	±	0.02	0.22		±	0.03	0.24		±	0.02
VT/Ti	1.713	±	0.18	2.265	±	0.38***	2.31	±	0.32	3.1	±	0.57***	3.35		±	0.67	4.85		±	0.82****
HRR	56.63	±	14.08	53.65	±	13.07	32.6	±	9.94	30.35	±	14.56	15.65		±	6.26	10.57		±	10.1
SV	92.36	±	14.83	140.4	±	24.18****	87.3	±	14.5	132.8	±	22.3****	85.81		±	13.32	126.4		±	18.53****
Power	155.56	±	39.09	151.04	±	46.90	250.00	±	39.53	291.46	±	34.12*	308.33		±	35.36	342.08		±	30.99**

Values are shown as mean ± standard deviation. t%: difference from VO2max in percentage; Rf: respiratory frequency; VT: tidal volume; VE: ventilation; IV: inspiratory volume; VO2: absolute oxygen consumption; VCO_2_: carbon dioxide production; RER: respiratory exchange ratio; VE/O2: ventilatory equivalent for oxygen; VE/CO_2_: ventilatory equivalent for carbon dioxide; VO2/kg: relative oxygen consumption; METs: metabolic equivalent of task; HR: heart rate; VO2/HR: oxygen pulse; PetO2: end tidal partial pressure of oxygen; PetCO2: end tidal partial pressure of carbon dioxide; FAT%: percentage of energy from fat; CHO%: percentage of energy from carbohydrate; Ti: inspiratory time; Te: expiratory time; Ttot: respiratory cycle duration; Ti/Ttot: inspiratory time divided by respiratory cycle duration; VD/VT: dead space; VT/Ti: ventilatory drive; HRR: heart rate reserve; SV: stroke volume. *p < 0.05; **p < 0.01; ***p < 0.001 and ****p < 0.0001.

### Multiple regression analysis and PCA

**FIGURE 2:**
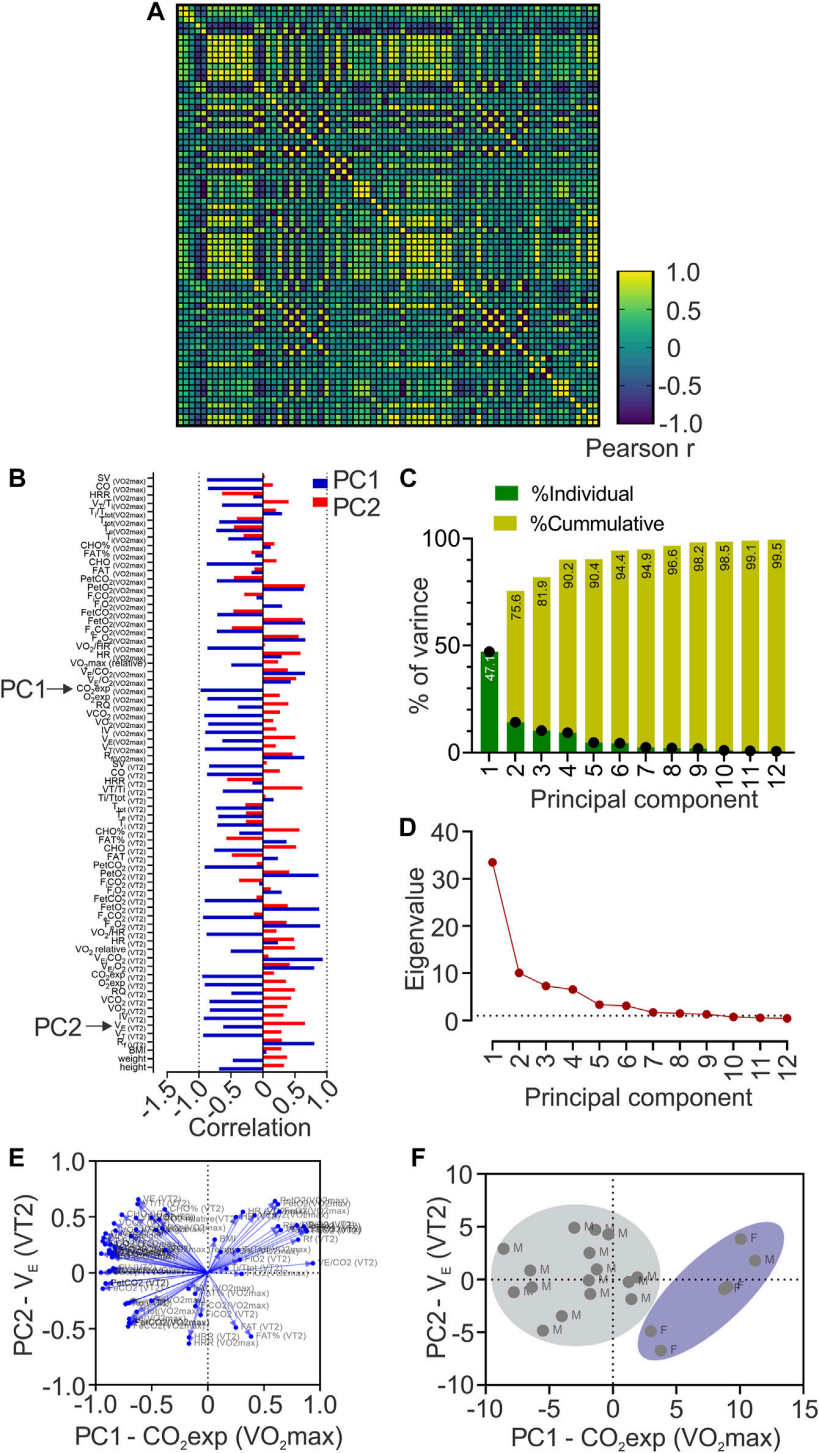
Principal components analysis (PCA). **(A)**, Heatmap of Pearson correlation coefficients between all data sets showing Pearson r values; **(B)**, Loadings of each to PC1 and PC2; **(C)**, Contribution of each Principal Component (PC) to the total variance of data; **(D)** Principal component analysis showing Eigenvalue higher than one in PC1 to PC6; **(E)**, Biplots are representing the contribution of each variable to the six first principal components; **(F)**, Biplot of plotted principal components scores.

To study the possible relationship of 72 cardiorespiratory, metabolic, and anthropometric variables during VT2 and VO2max (related performance variables) with the COP among the sex of individuals, we computed Pearson R-values for every pair of values in a multivariable correlation matrix (Figure 2A). Our analysis revealed that COP was related, positive and negative, with several cardiorespiratory variables (Figures 2A, B).

PCA revealed 12 principal components; however, only PC1 to PC6 were considered as significant (eigenvalues higher than 1, Figures 2C, D) and explained 94.4% of the total variance of data (Figure 2C). Nevertheless, PC1 contributed 47.1%, and PC2 to 28.5% of the total variance (Figure 2C). Loadings (or the contribution of variable “m” to PCn) of each variable to PC1 and PC2 is depicted in Figure 2D and we also generated biplots showing the loadings of each variable to the six first PCs (Figure 2E), based on Pearson-r values of between variables at PC1 and PC2 (Supplementary Table S3), revealing that CO_2_exp at VO2max majorly contributed to PC1; and VE at VT2, to PC2 (Figure 2B). We plotted PC scores in a biplot, depicting all individuals distinguished by sex (Figure 2F).

## Discussion

The purpose of the study was to resolve the determinants of COP in highly trained athletes and its influence on maximum and sub-maximum variables during CPET through PCA. The main findings of this study were: i) the COP was located before VT1; ii) PCA reveals that COP could influence CO_2_exp at VO2max (PC1) and VE at VT2 (PC2). Therefore, these results strongly suggest that COP influences cardiorespiratory performance-related variables at VT2 and VO2max. Further, it is possible that during long-term cessation training, due to, i.e., injury, the COP could be considered used during non-maximal CPET to estimate how much the athlete has been affected by the stop of training, suggesting that the COP could be used during rehabilitation periods.

### COP characteristics

The VE/VO2 minimum value (i.e., COP) could be considered a submaximal calculation for the best integration between the circulatory and pulmonary systems (Ramos et al., 2012). Ramos and Araujo (2017) conducted a study that included 3,331 subjects with and without chronic diseases, where they showed three COP categories, defined by the cut-off values < 22, 22-30, and >30 VE/VO2 (Ramos and Araújo, 2017). Importantly, when COP is > 30, it is a good predictor of all-cause mortality independently or in combination with lower VO2max, compared to those with <22 value (Ramos and Araújo, 2017). Our data revealed that COP was located before VT1 and VT2, consistent with previous reports in the non-athlete population (Ramos et al., 2012) and in professional soccer players (Silva et al., 2018). Importantly, our results depicted that both female and male athletes showed values between 22 and 30 VE/VO2 ratio, which was a cutoff classified as moderate, evidencing a good interaction between circulatory and respiratory function (Ramos and Araújo, 2017). However, although this sex-related difference is accordingly to previous reports, its dissimilarity only has been shown in a non-athlete population. Then, our data suggest that these sex-related differences could be transversal, independent of the training regimen.

The VE/VO2 values have been previously used to describe the oxygen efficiency uptake (OUE) since OUE = 1000/VE/VO2 (Sun et al., 2012a; Sun et al., 2012b). The oxygen efficiency uptake plateau (OUEP) corresponds to a 90-s average of the highest consecutive measurements from VO2/VE (Sun et al., 2012a; Sun et al., 2012b). Sun and colleagues (2012) showed that OUEP is a good predictor of early death in patients with heart failure (Sun et al., 2012b). Further, the oxygen uptake efficiency at the anaerobic threshold, which can be obtained from a 60-s average of OUE, was similar and highly associated with OUEP (Sun et al., 2012a; Sun et al., 2012b; Sheridan et al., 2021). Nevertheless, OUEP does not accurately predicts VO2max in non-athletes (Sheridan et al., 2021). Likewise, a recent study reported no correlation between OUEP and VO2max in runners even after an increase in OUE (Jost et al., 2022). Despite the previously mentioned absence of correlation between OUEP, OUE at anaerobic threshold (or other values through VE/VO2 signal) with performance variables, such as VO2max, physiologically VO2 as well as VE both are influenced by cardiorespiratory and peripheral variables (Sun et al., 2012a; Sun et al., 2012b) Therefore, we used PCA to show how COP, a simplified index to obtained from the lowest VE/VO2 ratio in 60 s, influences metabolic and cardiorespiratory variables at VO2max and VT2 in the next section.

### Contribution of principal components to COP in endurance athletes

During a CPET, there are several measures and calculated variables (cardiorespiratory and metabolic); however, despite that this would be considered an advantage, at the same time, it could also generate confusion. Currently, we evaluate 72 variables (cardiorespiratory and metabolic) derived from CPET, and we used PCA to determine what variables were more related to COP variance. PCA revealed that PC1 and PC2 explain 75.6% of the total COP variance. The principal variables that mainly explain the PC1 and PC2 were CO_2_exp at VO2max and VE at VT2, respectively. Mechanistically, during CPET, the CO_2_ production is related to metabolic acidosis, which is compensated by hyperventilation, reflected by an increase of VE (Nicolò et al., 2020a). Our data revealed that CO_2_exp at VO2max mainly explains the COP variance (PC1: 47.1%); however, we found that CO_2_exp at VO2max has shown a negative correlation with COP. Accordingly, this negative association could potentially support that lower COP allows better metabolic compensation at VO2max and could be relevant considering that world-class skiers showed two times a longer plateau at VO2max compared to national-class skiers (Sandbakk et al., 2011). Besides, several cardiopulmonary variables relevant to reaching high VO2max in endurance athletes are loading PC1 (Figure 2B) (Midgley et al., 2007). Indeed, VO2max is mainly limited by the stroke volume (SV) in well-trained athletes (Ouellet et al., 1969). Then, it is possible to propose that a lower COP value could be related to a better performance (negatively correlated with SV at VO2max). In addition, despite the lower number of female participants, we found that female subjects formed a cluster, which reflects that CO_2_exp at VO2max (PC1), could be more relevant to females than male participants to explain the COP variance.

Principal component results revealed that PC2 explained 28.5% of total COP variance, explained mainly by VE at VT2. In addition, we found that VE at VT2 was negatively correlated with COP. It has been proposed that ventilatory response to exercise is critical to maintaining endurance performance (Tiller, 2019; Nicolò et al., 2020a; Nicolò et al., 2020b). Indeed, elite endurance athletes reach <75% VO2max during long-term time-trial running, evidencing that running a marathon reaches moderate to high VE (Joyner and Coyle, 2008). Higher VE is necessary to maintain altered homeostasis when CO_2_ production increases (Ghosh, 2004). Moreover, it has been evidenced that one of the training adaptations in elite cyclists results in an increased VE at VT2 (Hoogeveen, 2000). In our study, those athletes with lower COP reached higher VE at VT2. Hence, PCA revealed that a low COP could help predict better cardiopulmonary variables for higher performance. It is essential to mention that VE is influenced by VT and Rf (Nicolò et al., 2020a); Rf has been demonstrated to be significant in sports and exercise (Nicolò et al., 2020b), and has been related to muscle fatigue (Marcora et al., 2008), rate of perceived exertion, and exercise tolerance (Nicolò et al., 2014; Nicolò et al., 2016; Nicolò et al., 2018), while VT increases to match VE (Nicolò et al., 2014; Nicolò et al., 2016; Nicolò et al., 2017; Nicolò et al., 2018). Therefore, our result suggests that COP could be associated with performance-related variables, which in turn suggests that the assessment of COP, obtained at submaximal intensity, could contribute to determining the competitive state of the athletes, at a low time-effort ratio. COP can be assessed likely without the interference of intensity distribution training since elite, and world-class athletes’ prominent characteristic is to perform at high volume at low-intensity (Sandbakk et al., 2011). In contrast, high intensity is prescribed at a lower volume to avoid chronic stress (Seiler, 2010; Stöggl and Sperlich, 2015). In addition, COP can possibly be helpful during short and long-term training cessation (i.e., injury) to estimate detraining. However, our study was not focused on injured athletes because first, we needed to find whether, on the same subject, the COP could be influenced by some performance-related variables. Then, in future research, it is crucial to determine the impact of short and/or long-term training cessation on COP and determine if it could help to predict how much the athlete has been affected, contributing to the return sport continuum in highly trained subjects. Finally, our findings can be useful in non-athlete population or with chronic disease, where COP assessment could help to determine which variables contained in PC1 and PC2 are being affected allowing clinicians to aim different approaches of treatment.

### Strength and limitations

Our study is not without limitations. Our sample was healthy athletes and not injured athletes, which could limit the interpretation of the results. However, it is essential to mention that, first, we need to find whether, in the same subjects, the COP could be influenced by some performance-related variables after moving to injured athletes. Furthermore, no blood samples were taken, which could contribute to a better understanding of the relationship between metabolic demands and oxygen consumption efficiency related to COP. Then, our results from PCA strongly support that COP could be influenced by VT2 and VO2max cardiorespiratory and metabolic variables; thus, this may help to support the notion of using the COP as a possible tool to track ventilatory thresholds to delineate exercise intensity domain and assess how cardiopulmonary and metabolic performance is affected by, i.e., short, and long-term training cessation.

In summary, PCA revealed that COP variance was mainly explained by CO_2_exp at VO2max and VE at VT2. Hence, COP could be an index capable of evaluating O2 consumption efficiency and tracking ventilatory thresholds at different exercise intensity domains due to its association with cardiopulmonary and metabolic variables, as well as performance-related variables. Thus, considering that COP can be obtained at submaximal intensity, our results strongly suggest that the COP calculation in these athletes could be used as a time-effort efficiency evaluation and monitoring tool during the off-season and competitive periods, as well as through rehabilitation and the return to sport continuum.

## Conclusion

Our data suggested that COP evaluation could provide crucial information about cardiorespiratory performance-related variables at VT2 and VO2max, allowing us to identify an area of improvement among described PC variables. Besides, exercise performance can be negatively affected due to short-term and long-term stop training, i.e., off-season, illness, injuries, or surgical procedures. Thus, COP could be helpful to track ventilatory thresholds or even be implemented in early rehabilitation to estimate the current cardiorespiratory performance of the athlete and their evolution during the sports reinstatement process without the need for a maximal test (i.e., CPET). In addition, PCA revealed that COP is closely related to cardiopulmonary variables at VT2 and VO2max, which supports its usefulness in determining actual performance.

## Data Availability

The raw data supporting the conclusion of this article will be made available by the authors, without undue reservation.

## Glossary

CPET: Cardiopulmonary exercise test
COP: Cardiorespiratory optimal point
VO2: Oxygen consumption
**VCO**_**2**_: Carbon dioxide output
VE: Minute ventilation
VE/VO2: Minute ventilation to oxygen consumption ratio
PCA: Principal Component Analysis
VO2max: maximal oxygen consumption
VT: ventilatory thresholds
VT1: Ventilatory threshold 1
VT2: Ventilatory threshold 2
PC: Principal component
PC1: expired CO_2_ at VO2max
PC2: VE at VT2
FEV1: Forced expiratory volume at first second
VC: Vital capacity
ECG: Electrocardiogram
PEF: Peak expiratory flow
PIF: Peak inspiratory flow
FEF 25: Forced expiratory flow at 25%
FEF 50: Forced expiratory flow at 50%
FEF75: Forced expiratory flow at 75%
W: Watts
O2: Oxygen
**CO**_**2**_: Carbon dioxide
N2: Nitrogen
FVC: Forced vital capacity
**CO**_**2**_ **exp**: Expired CO_2_
